# Supplementary material for: A Survey to Understand Parent/Caregiver and Children’s Views on Devices Used for the Administration of Oral Pediatric Medicines in Japan
Source: Children (Basel). 2022 Feb 3;9(2):196. doi: 10.3390/children9020196 (PMC8869804; doi:10.3390/children9020196)
Supplement: Supplementary file 1 [file children-09-00196-s001.zip › 20211228Supplementary materials.pdf]

Figure S1. The proportion of devices used for taking each type of medicine.

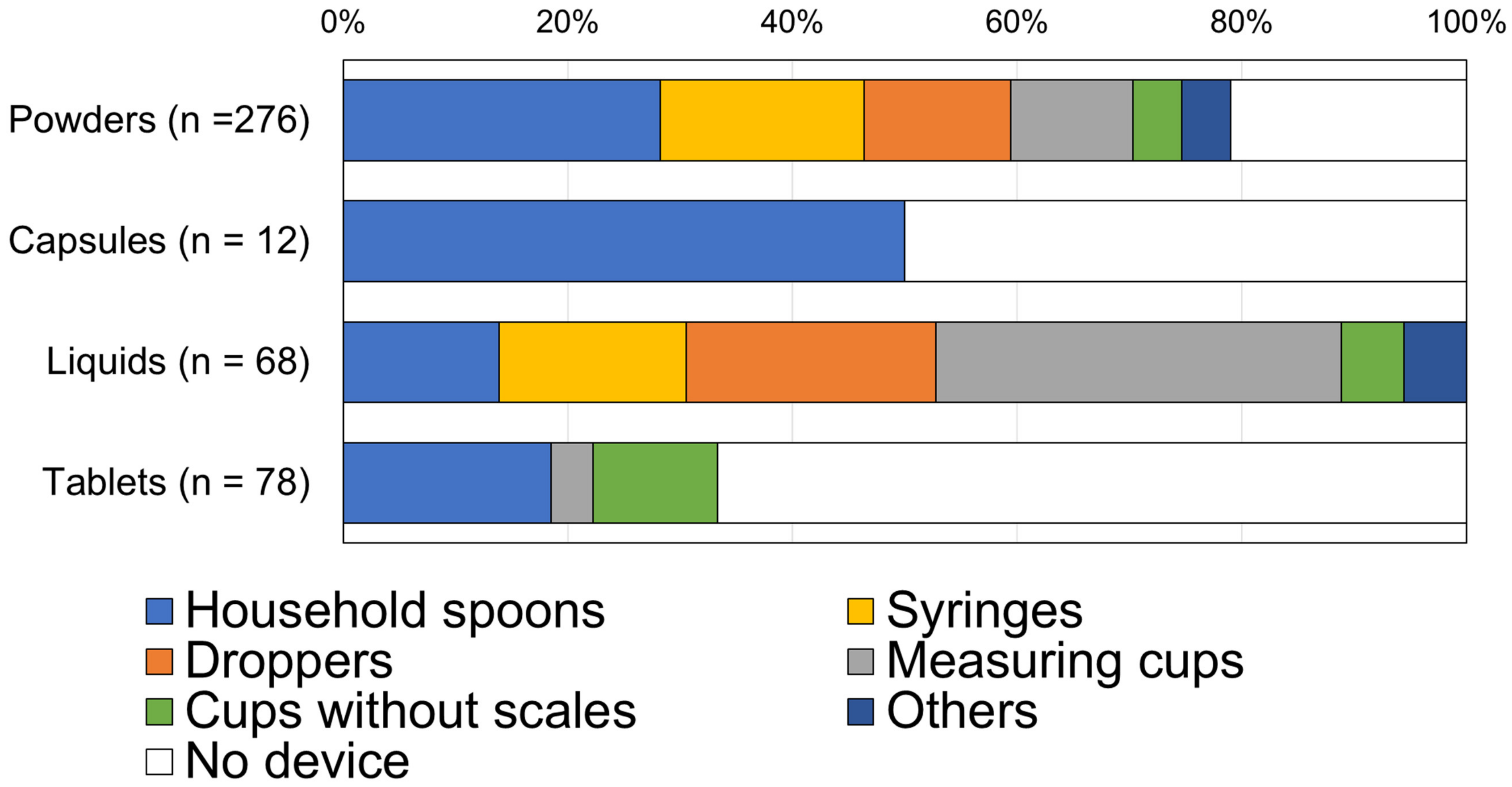

Figure S2. The user-friendliness of each device by age**.**

(a) Less than 12 months

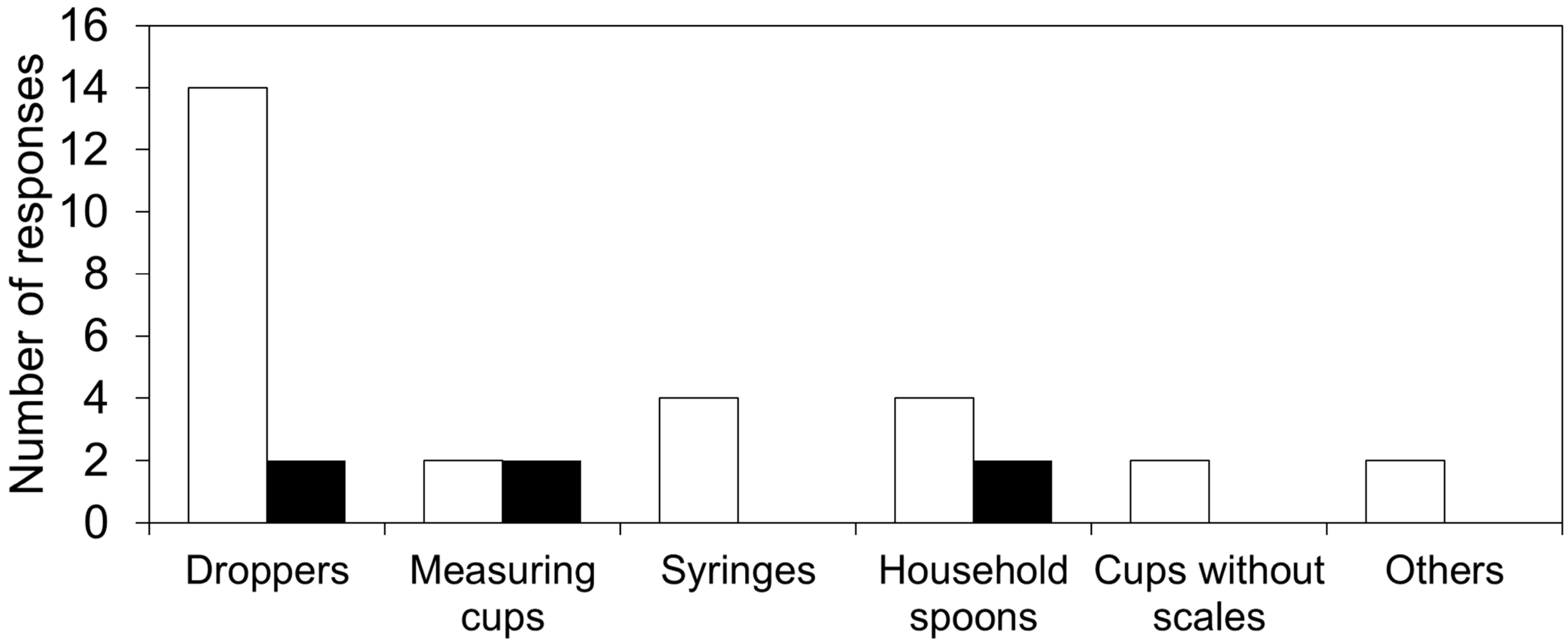

Opened boxes indicated the number of answers for "Easy to use", and closed boxes indicated the number of answers for "Difficult to use".

(b) 12 to 23 months

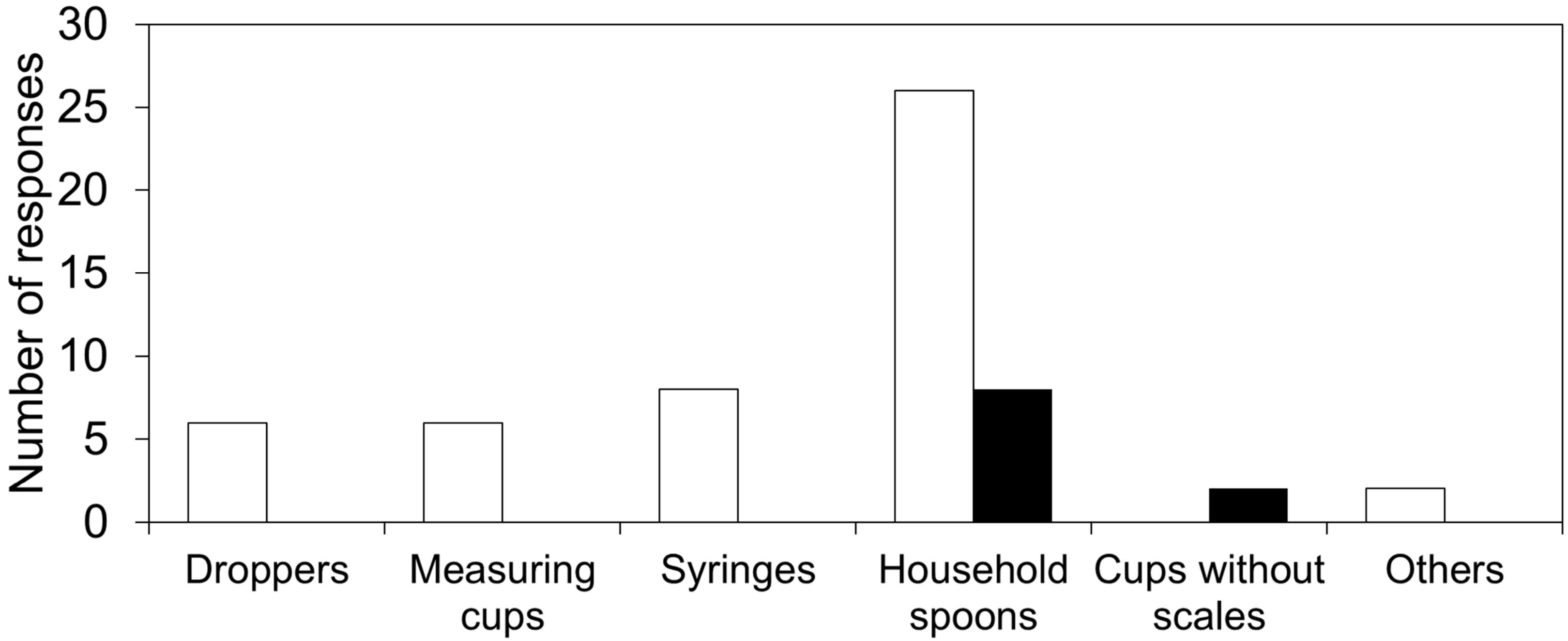

Opened boxes indicated the number of answers for “Easy to use”, and closed boxes indicated the number of answers for “Difficult to use”.

(c) 2 to 5 years old

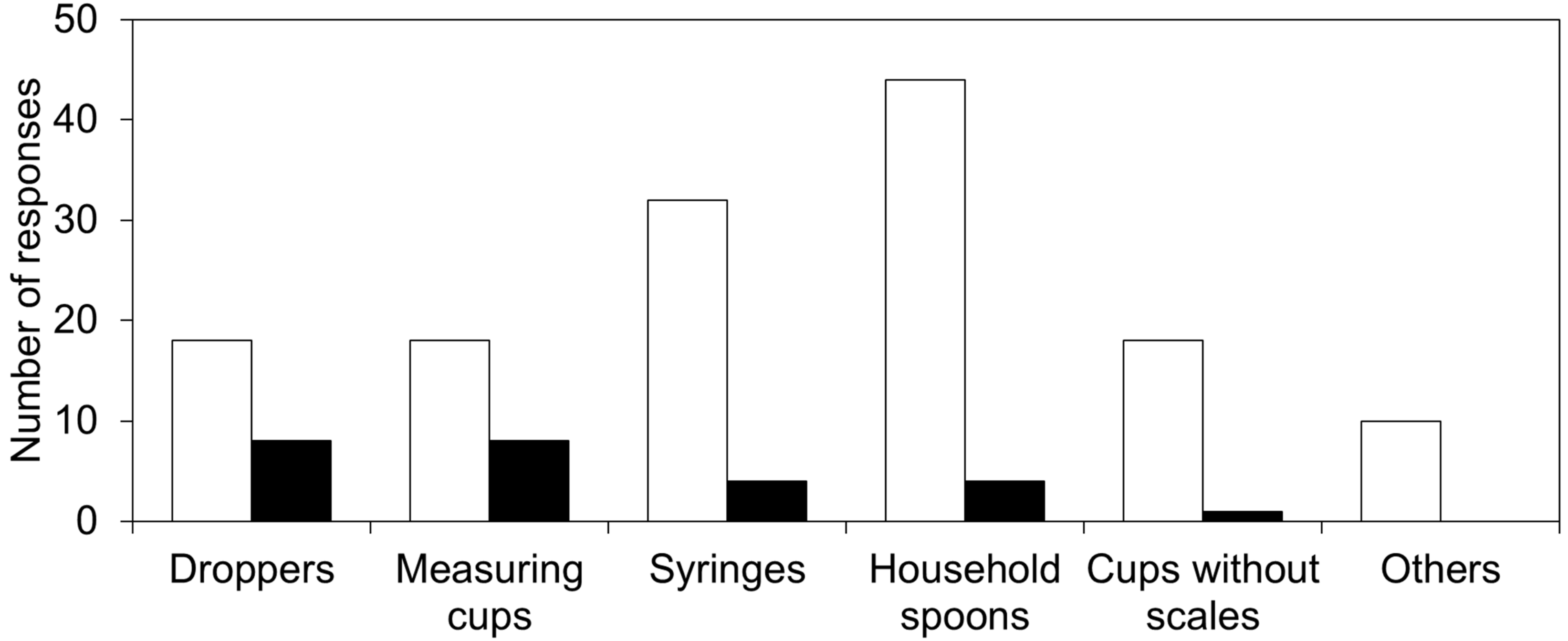

Opened boxes indicated the number of answers for “Easy to use”, and closed boxes indicated the number of answers for “Difficult to use”.

(d) 6 to 9 years old

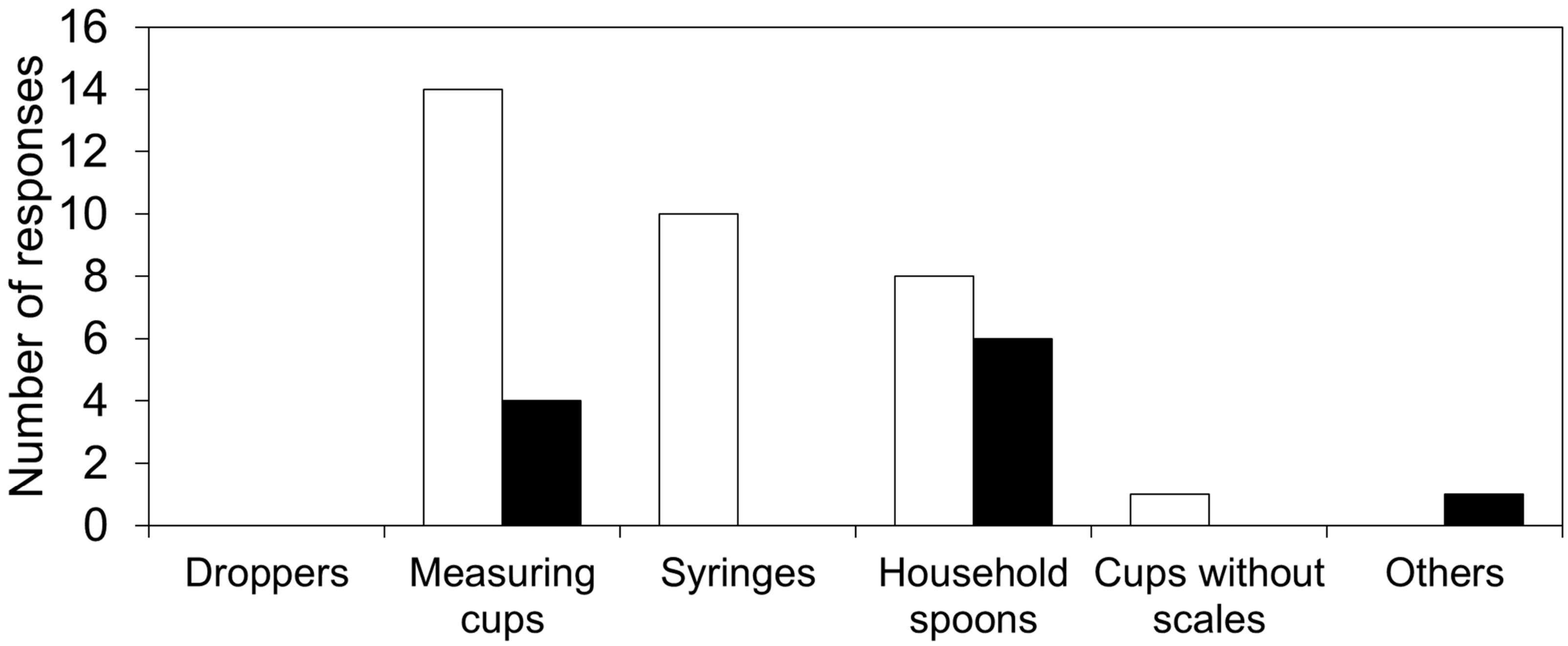

Opened boxes indicated the number of answers for “Easy to use”, and closed boxes indicated the number of answers for “Difficult to use”.

(e) 10 to less than 18 years old

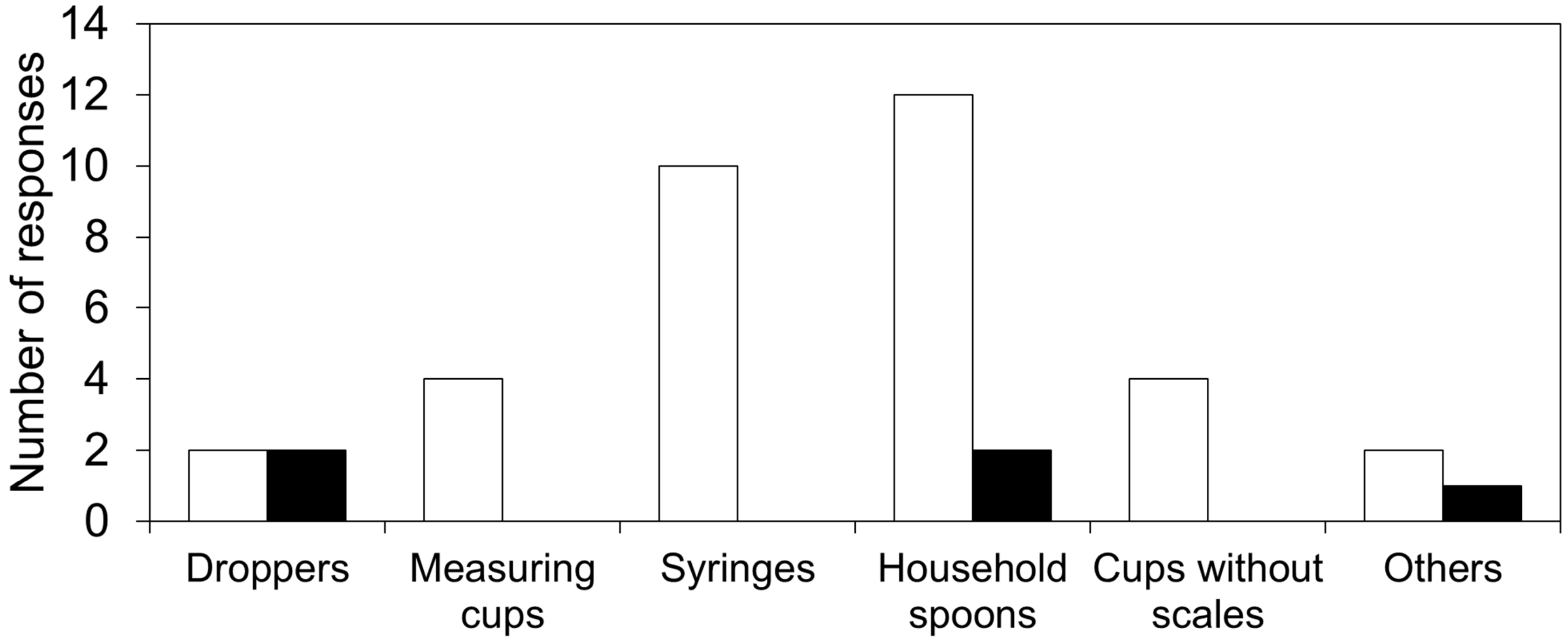

Opened boxes indicated the number of answers for “Easy to use”, and closed boxes indicated the number of answers for “Difficult to use”.

Figure S3. The user-friendliness of each device for liquid administration.

(a) Less than 12 months

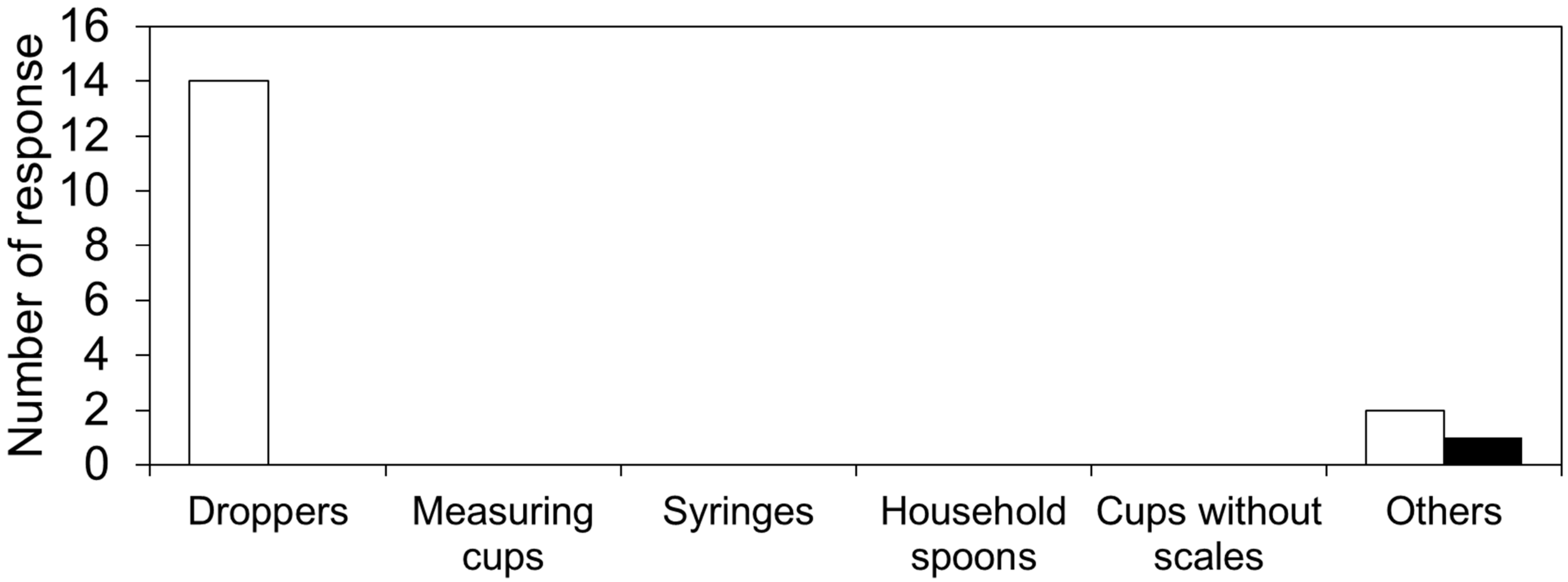

Opened boxes indicated the number of answers for “Easy to use”, and closed boxes indicated the number of answers for “Difficult to use”.

(b) 12 to 23 months

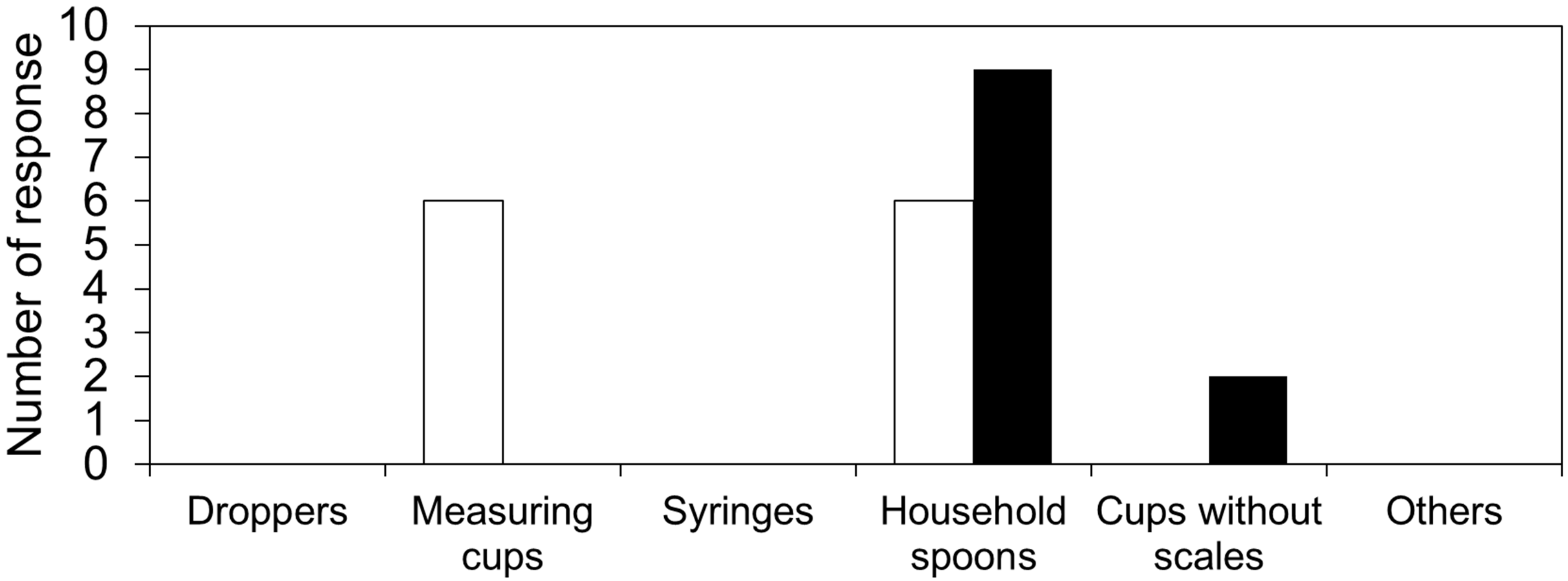

Opened boxes indicated the number of answers for “Easy to use”, and closed boxes indicated the number of answers for “Difficult to use”.

(c) 2 to 5 years old

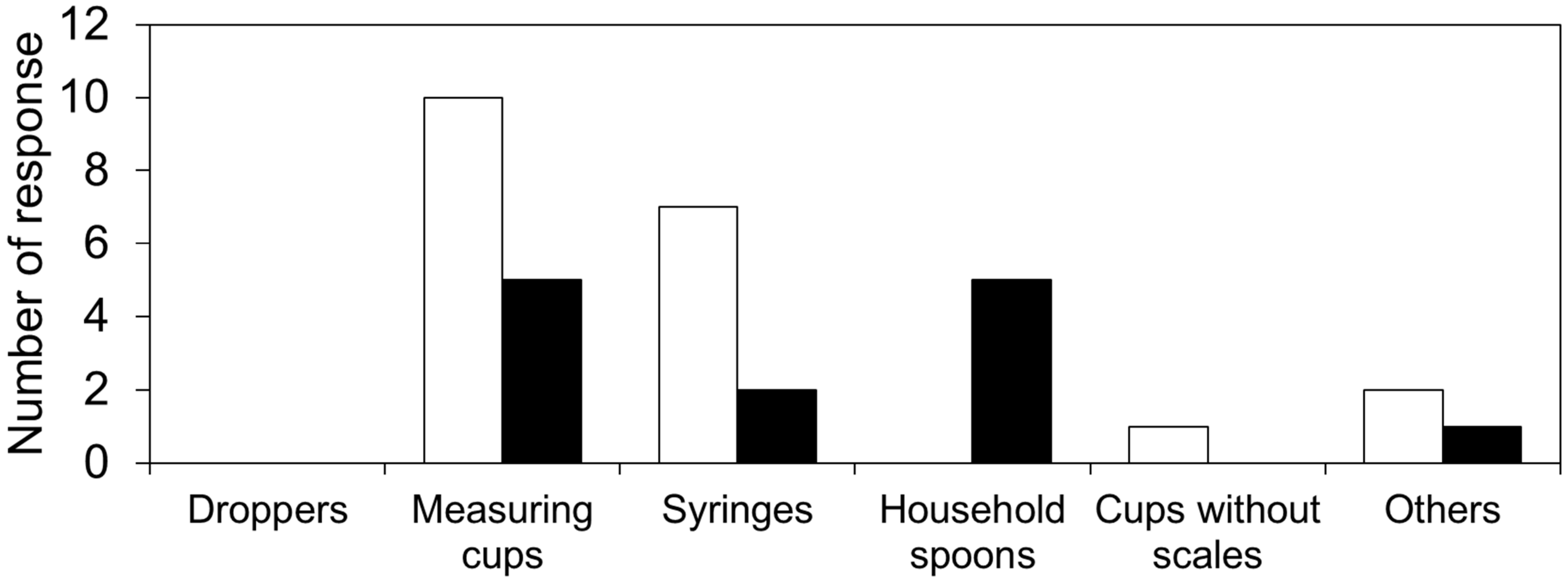

Opened boxes indicated the number of answers for “Easy to use”, and closed boxes indicated the number of answers for “Difficult to use”.

(d) 6 to 9 years old

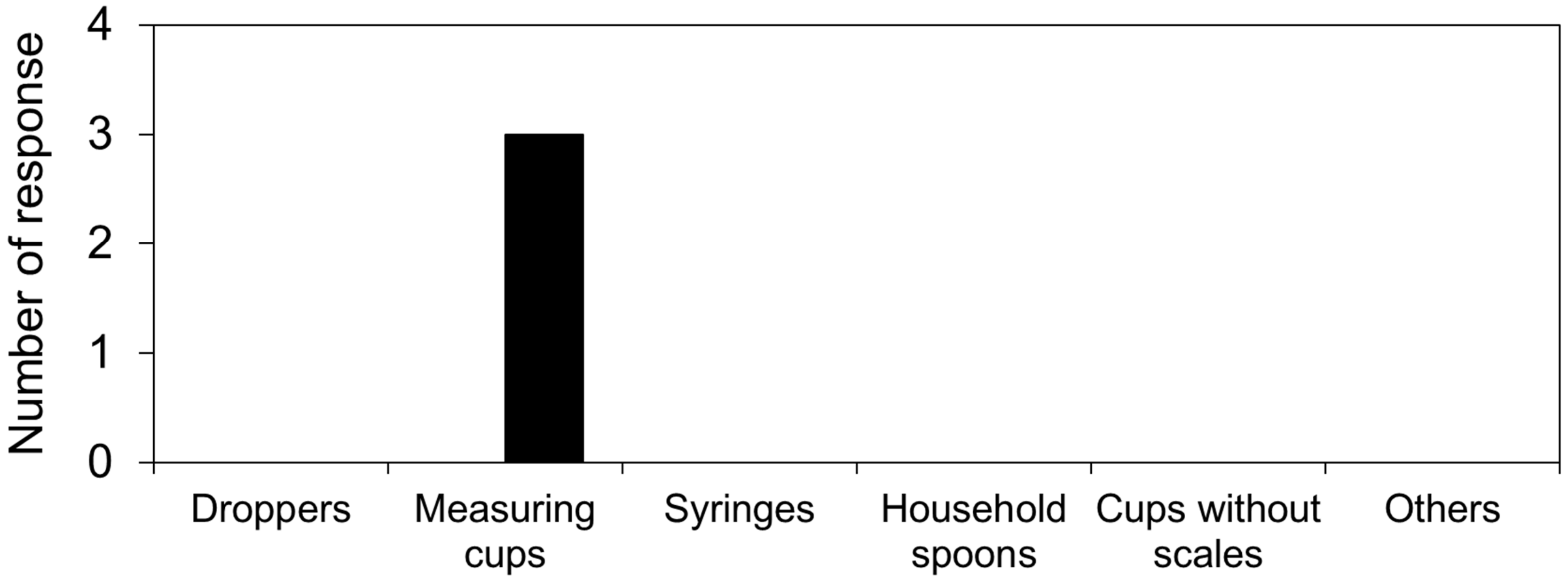

Opened boxes indicated the number of answers for “Easy to use”, and closed boxes indicated the number of answers for “Difficult to use”.

(e) 10 to less than 18 years old

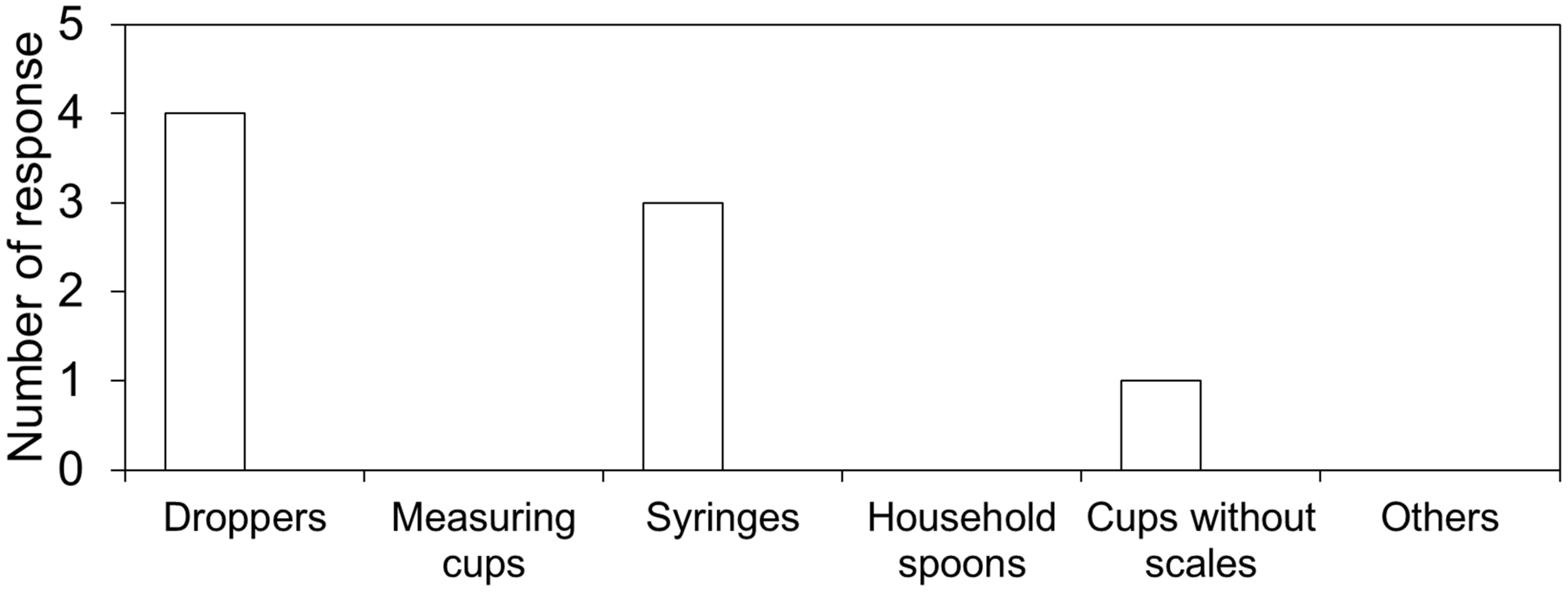

Opened boxes indicated the number of answers for “Easy to use”, and closed boxes indicated the number of answers for “Difficult to use”.

Figure S4. The user-friendliness of each device for powder administration.

(a) Less than 12 months

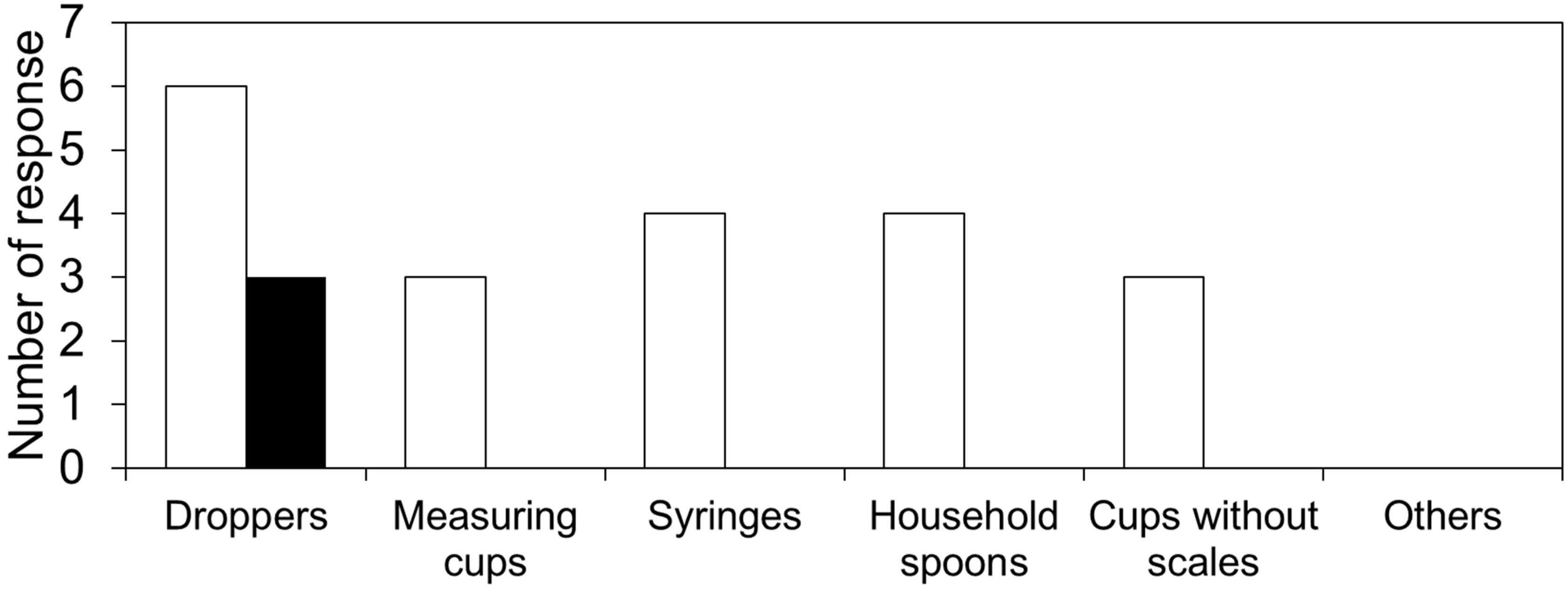

Opened boxes indicated the number of answers for “Easy to use”, and closed boxes indicated the number of answers for “Difficult to use”.

(b) 12 to 23 months

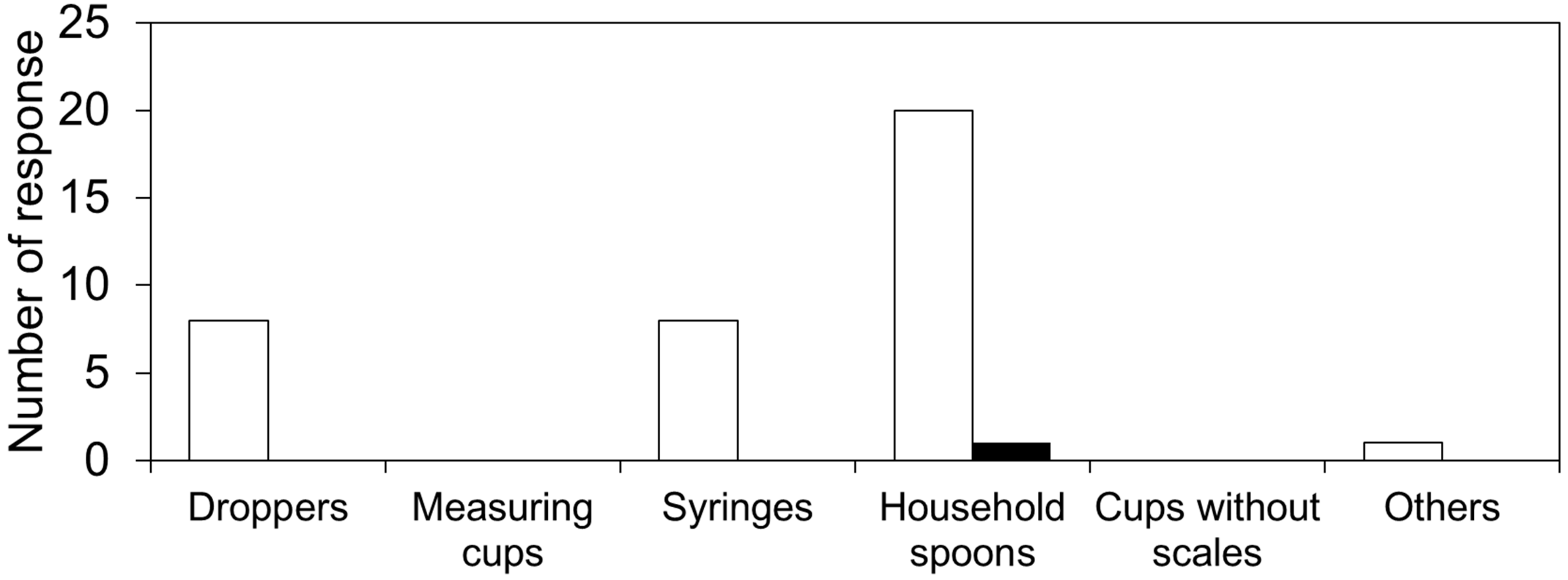

Opened boxes indicated the number of answers for “Easy to use”, and closed boxes indicated the number of answers for “Difficult to use”.

(c) 2 to 5 years old

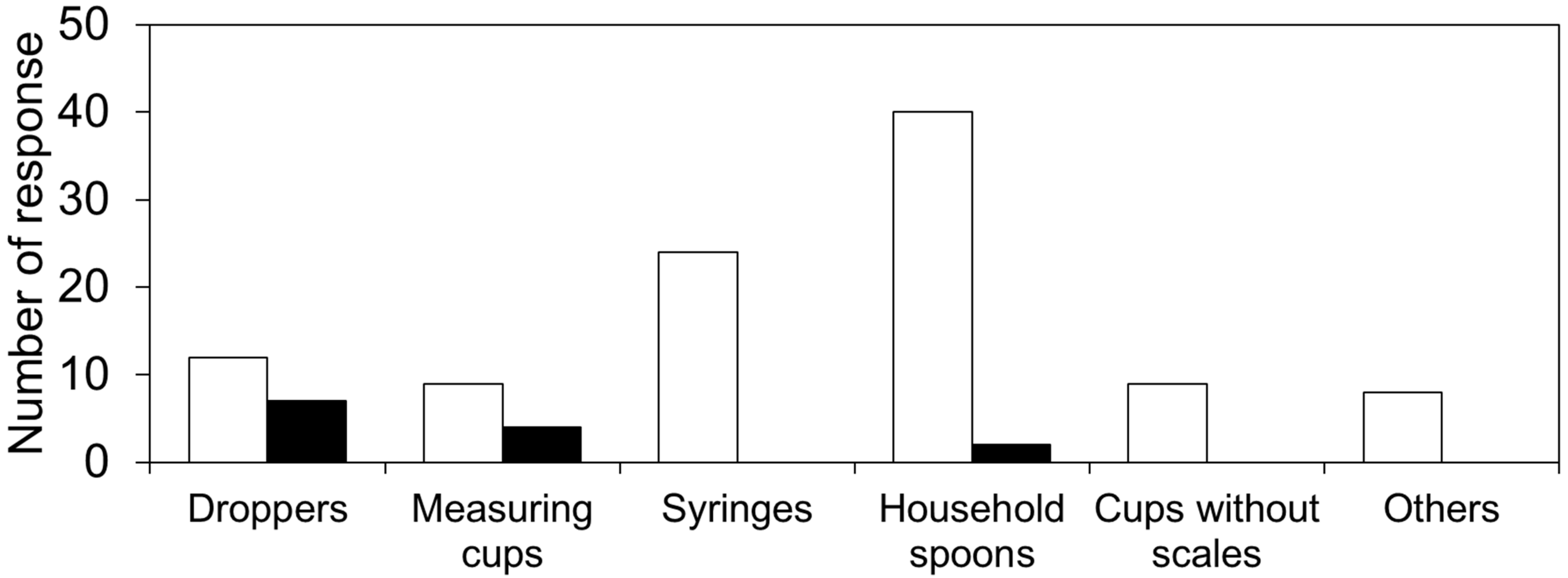

Opened boxes indicated the number of answers for “Easy to use”, and closed boxes indicated the number of answers for “Difficult to use”.

(d) 6 to 9 years old

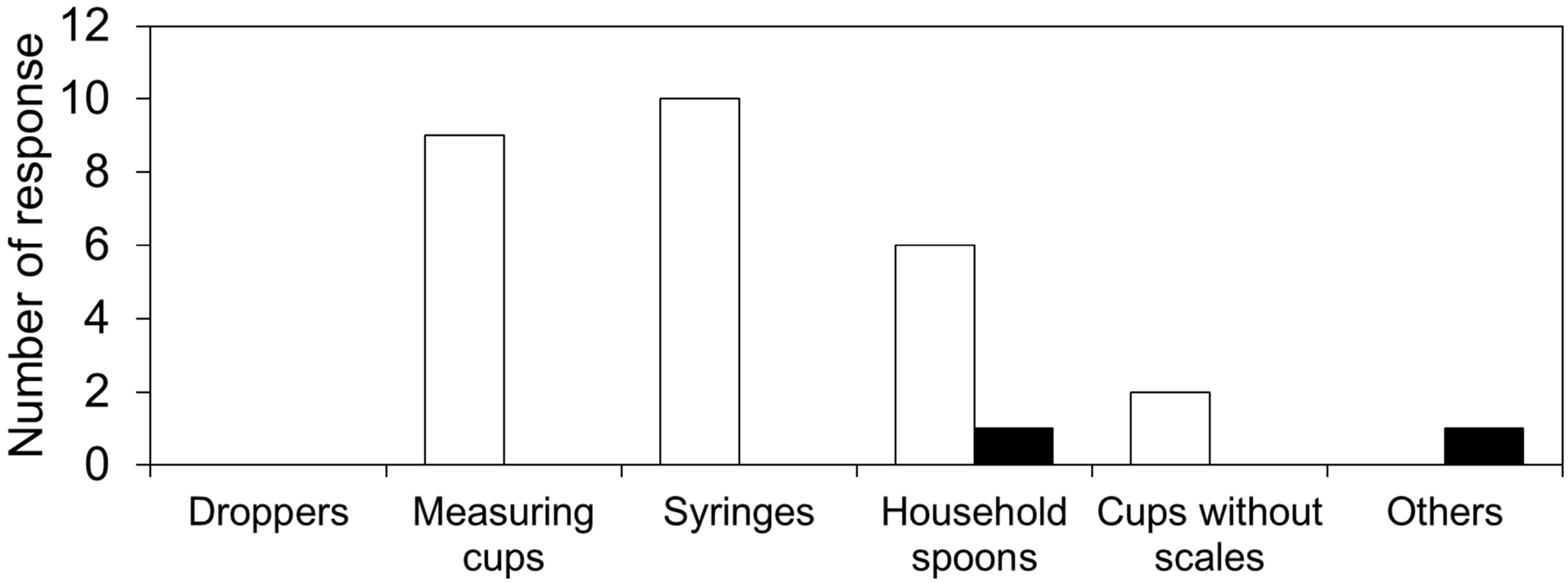

Opened boxes indicated the number of answers for “Easy to use”, and closed boxes indicated the number of answers for “Difficult to use”.

(e) 10 to less than 18 years old

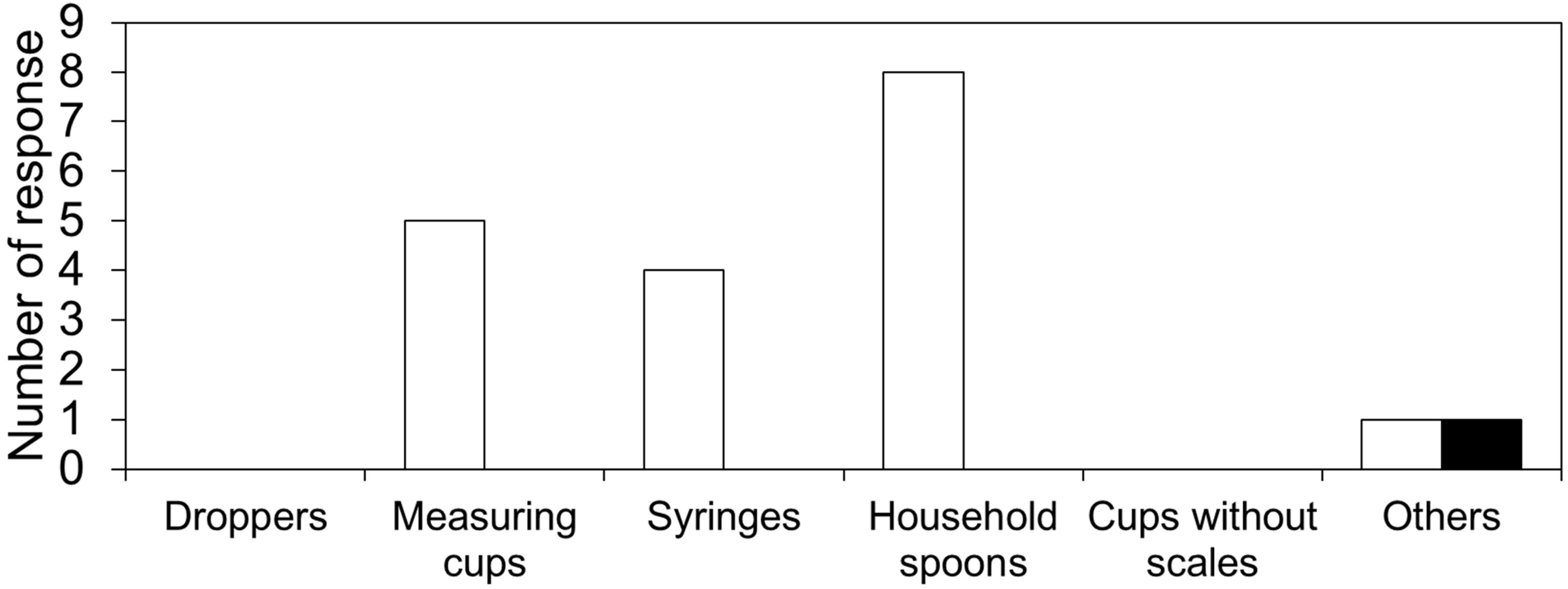

Opened boxes indicated the number of answers for “Easy to use”, and closed boxes indicated the number of answers for “Difficult to use”.

Table S1. Questionnaire items.

Questionnaire items	Options
(#1) Age of the child	(i) Less than 12 months<br>(ii) 12 to 23 months<br>(iii) 2 to 5 years old<br>(iv) 6 to 9 years old<br>(v) 10 to less than 18 years old
(#2) Relationship with the child who takes oral medicine	(i) Parent(s)<br>(ii) Grandparent (s)<br>(iii) Other family members (s)<br>(iv) Other caregiver(s)
(#3) Type of oral medicine the child has taken most recently	(i) Tablets (including oral disintegrating tablets)<br>(ii) Drops<br>(iii) Capsules<br>(iv) Liquids (including syrups and suspensions)<br>(v) Powders (including fine granules, granules, and dry syrups)
(#4) Device used for the selected medicine	(i) Household spoon<br>(ii) Measuring cup<br>(iii) Measuring spoon<br>(iv) Oral syringe<br>(v) Dropper<br>(vi) Cups (with no scale)<br>(vii) Others (please specify)<br>(viii) No device was used
(#5) Duration of the selected dosing device use	(i) Within 1 week<br>(ii) 1 to 2 weeks<br>(iii) 3 to 4 weeks<br>(iii) 1 to 11 months<br>(iv) Others (free description)<br>(v) More than 1 years<br>(vi) Unknown
(#6) Frequency of the selected device using	(i) Once a day

		(ii)	Twice a day
		(iii)	Three times a day
		(iv)	Four times a day
		(v)	Other (please specify)
(#7)	Friendliness of the selected device to use	(i)	Easy to use
		(ii)	Difficult to use
		(iii)	Neither easy nor difficult
(#8)	Instruction on how to use the device (who provide and how clear the instruction)		
	(#8-1) Whether were the instructions on how to use the device	(i)	Yes
		(ii)	No
		(iii)	Don't know
	(#8-2) Who gave the instructions to use the devices	(i)	Physician
		(ii)	Nurse
		(iii)	Pharmacist
		(iv)	Patient Information Leaflet (PIL)
	(#8-3) How clear the instructions were to follow	(i)	Clear
		(ii)	Not clear
		(iii)	Don't know
