# Supplementary material for: A Survey to Understand Parent/Caregiver and Children’s Views on Devices Used for the Administration of Oral Pediatric Medicines in Japan
Source: Children (Basel). 2022 Feb 3;9(2):196. doi: 10.3390/children9020196 (PMC8869804; doi:10.3390/children9020196)
Supplement: Supplementary file 1 [file children-09-00196-s001.zip › Supplementary fileS1.pdf]

西暦　2021年04月19日

研究実施許可書

研究責任者　齊藤 順平　殿

国立成育医療研究センター　理事長
五十嵐　隆

西暦2021年04月06日に貴殿から審査申請のあった研究を厳正に審査し、研究実施を許可します。

受付番号	2021-008
研究課題名	保護者および小学4年生以上の小児を対象とした、小児薬物療法に用いられる器具の使いやすさに関するアンケート調査～多施設・国際共同・前方視的・観察研究～
審査事項	■研究等の実施の適否<br>□研究等継続の適否<br>□重篤な有害事象<br>□研究に関する変更<br>□倫理的妥当性・科学的合理性を損なう事実報告<br>□研究の実施の適正性・研究結果の信頼を損なう事実報告<br>□その他
判定	■承認　□条件付承認　□継続審査　□非該当　□不承認<br>□研究継続　□条件付研究継続　□研究中止
備考	（条件付き承認の場合の研究実施許可条件）
